# Book Reviews

**Published:** 1897-01

**Authors:** 


### BOOK REVIEWS.

Reference-Book of Practical Therapeutics. By various authors. Edited by Frank P. Foster, M.D., editor of the *New York Medical Journal* and of Foster's Encyclopedic Dictionary. In two volumes. D. Appleton & Company, New York. Vol. I. pp. 652.

The first volume of this handbook, which has just appeared, has been written by over thirty well-known American authorities on therapeutics. The range of the book is not limited to the action of "drugs," but includes curative measures of all descriptions outside of surgical methods, such as the use of diets, exercise, massage, climate, heat and cold, hydriatics, electricity, condensed and rarefied air, etc. The trade names of chemical combinations used in medicine are also given. Each subject is treated according to its importance; and may be found quickly on account of the alphabetical arrangement and prominent type of the headings.

For the busy practitioner especially who desires to have at hand a full and correct account of all known therapeutical methods and the most recent drugs, this book will certainly prove an important contribution to his library. The authors are to be congratulated on the appearance of this volume, and the publishers have most satisfactorily done their part. A. W. S.

---

We acknowledge receipt from Dr. J. T. Eskridge, Denver, Colorado, of brochure containing a "Series of Articles on Speech-Defects as Localizing Symptoms, from a Study of Six Cases of Aphasia," reprinted from the *Medical News*, June 6th to September 19th, 1896. The cases make a very interesting collection of brain lesions, with a careful description of the symptoms and treatment in each case. It is an instructive contribution to the cerebral localization of certain functions, which will be of special interest to the neurologist and brain-surgeon.

